# Predictors of Permanent Pacemaker Implantation in Patients After Transcatheter Aortic Valve Replacement in a Chinese Population

**DOI:** 10.3389/fcvm.2021.743257

**Published:** 2022-01-06

**Authors:** Jiaqi Zhang, Chengwei Chi, Simiao Tian, Shulong Zhang, Jihong Liu

**Affiliations:** ^1^School of Graduate Studies, Dalian Medical University, Dalian, China; ^2^Heart Center, Affiliated Zhongshan Hospital of Dalian University, Dalian, China; ^3^Department of Scientific Research, Affiliated Zhongshan Hospital of Dalian University, Dalian, China

**Keywords:** aortic stenosis, transcatheter aortic valve replacement, complete atrioventricular block, permanent pacemaker, predictors

## Abstract

**Background:** Permanent pacemaker (PPM) implantation is the main complication of transcatheter aortic valve replacement (TAVR). Few studies have evaluated the requirement for PPM implantation due to ECG changes following TAVR in a Chinese population.

**Objective:** Our study aimed to evaluate the incidence and predictors of PPM implantation in a cohort of Chinese patients with TAVR.

**Methods:** We retrospectively evaluated 39 consecutive patients with severe native aortic stenosis referred for TAVR with a self-expandable prosthesis, the Venus A valve (Venus MedTech Inc., Hangzhou, China), from 2019 to 2021 at the Heart Center of Affiliated Zhongshan Hospital of Dalian University. Predictors of PPM implantation were identified using logistic regression.

**Results:** In our study, the incidence of PPM implantation was 20.5%. PPM implantation occurs with higher risk in patients with negative creatinine clearance (CrCl), dyslipidemia, high Society of Thoracic Surgeons (STS) Morbimortality scores, and lead I T wave elevation. TAVR induced several cardiac electrical changes such as increased R wave and T wave changes in lead V5. The main independent predictors of PPM implantation were new-onset left bundle branch block (LBBB) (coef: 3.211, 95% CI: 0.899–7.467, *p* = 0.004) and lead I T wave elevation (coef: 11.081, 95% CI: 1.632–28.083, *p* = 0.016).

**Conclusion:** New-onset LBBB and lead I T wave elevation were the main independent predictors of PPM implantation in patients undergoing TAVR. Clinical indications such as negative CrCl, dyslipidemia, high STS Morbimortality scores, and an increased T wave elevation before TAVR should be treated with caution to decrease the need for subsequent PPM implantation.

## Introduction

Aortic valve stenosis (AVS) has become the most prevalent acquired heart valve disease pathology (1). Transcatheter aortic valve replacement (TAVR) has been proven to be an efficient treatment for patients with severe AVS. Patients who suffer from this disease are at high-to-intermediate surgical risk (2–4). More recently, new randomized trials have broadened the clinical indications for the procedure, with its efficacy in intermediate and low-risk patients also being demonstrated (2–5). There has been a high demand for TAVR since its introduction in China. These figures are expected to exponentially increase due to the increasing age of the population (6).

This is timeous given that the first TAVR procedure has was performed in China in 2010. To date, only 3,500 patients across approximately 100 hospitals have received TAVR. Currently, self-expandable, mechanically expandable, and balloon-expandable aortic valves are clinically used in TAVR procedures in Western countries. Whereas the majority of TAVR cases in China involve self-expandable valves, investigations into the outcome of these valves in patients with AVS are lacking.

With the development of new-generation valves and operating methods, the occurrence of redo heart valve replacement, paravalvular leakage, and blood vessel complications has significantly decreased. Nevertheless, subsequent permanent pacemaker (PPM) implantation on account of complete atrioventricular block (AVB) is one of the most common complications after TAVR as diagnosed by ECG (7).

Therefore, the purpose of our study was to compare the type and frequency of ECG changes before and after TAVR and at one-month follow-up after discharge. We investigated these ECG changes to determine the main predictors of conduction disorders that lead to PPM implantation after TAVR in Chinese patients with self-expandable valves.

## Methods

### Study Population

We retrospectively evaluated 47 consecutive patients with severe native aortic stenosis referred for TAVR with the self-expandable Venus A valve (Venus Med Tech Inc., Hangzhou, China) from 2019 to 2021 at the Heart Center of Affiliated Zhongshan Hospital of Dalian University. The exclusion criteria included patients with PPM prior to TAVR (*n* = 2), intraoperative mortality (*n* = 1), valve-in-valve procedures (*n* = 3), and patients where 12-lead ECGs were unavailable (*n* = 2). Considering this, only 39 patients were included in the study. Surgical indications of patients were assessed and decided upon by a multidisciplinary team of doctors consisting of cardiac surgeons, cardiologists, echocardiologists, and anesthetists. A self-expandable aortic valve was deployed using the transfemoral approach in all patients. The study was approved by the ethics committee of Affiliated Zhongshan Hospital of Dalian University. The study also complied with the Declaration of Helsinki. All patients who participated in the study signed written informed consent forms.

### Electrocardiogram Analysis

All patients underwent immediate standardized (10 mm = 1 mV, 25 mm/s) 12-lead ECG before and one month post-operation with subsequent retrospective analysis of the data. Parameters included heart rate, rhythm, axis deviation, PR interval, type of AVB, QRS interval, type of bundle-branch block, QT interval, and corrected QT interval (cQT).

### Statistical Analysis

Categorical variables are expressed as numbers and percentages. All continuous data were expressed as means ± standard deviation (SD) or medians ± range as appropriate. Categorical variables were compared using Chi-squared or Fisher exact tests. Continuous variables were compared with the Student's *t-*test (2-tailed) or Wilcoxon signed-rank tests as appropriate. To identify predictors of patients with PPM dependency after TAVR, logistic regression, with Firth's correction due to the small sample size, was performed. All analyses were conducted using R (version 3.2.2; R Foundation for Statistical Computing, Vienna, Austria) or SAS Statistics (version 9.4; North Carolina, America). A *p*-value < 0.05 was considered statistically significant.

## Results

The detailed clinical baseline characteristics of patients in total and with or without PPM implantation are shown in Table 1. The mean age of the 39 consecutive patients was 75.0 ± 8.6 years, and 53.8% of the study population was male. Nearly 70% of the patients with severe AVS were at intermediate or high surgical risk with New York Heart Association (NYHA) classification III/IV. The average EuroScore II and Society of Thoracic Surgeons (STS) Mortality scores were 7.97 ± 8.53 and 3.18 ± 2.81, respectively. No statistical differences were observed between the two groups with the exception of higher dyslipidemia and worse creatinine clearance (CrCl) in the group with PPM implantation. The STS Morbimortality scores at baseline was much higher among the patients with PPM implantation than among those without PPM implantation (with PPM 20.93 ± 8.72 vs. without PPM 13.96 ± 7.20, *p* = 0.025). Complete AVB (62.5%) was the main indication for patients with PPM implantation after TAVR. In our study, the incidence of PPM implantation was 20.5%. Among patients with PPM implantation within 30 days of undergoing the TAVR procedure, the mean time to PPM implantation after TAVR was 5.8 ± 2.8 days.

**Table 1:** Baseline characteristics according to the group of study population: Total, without permanent pacemaker (PPM) and with PPM.

	**Total (*n =* 39)**	**Without pacemaker (*n* = 31)**	**With pacemaker (*n* = 8)**	***p* value**
Age, years	75.0 ± 8.6	74.3 ± 9.1	77.9 ± 6.0	0.293
Male gender	21 (53.8)	17 (54.8)	4 (50)	0.807
Heart Failure, NYHA Class I/II	3 (7.7)/8 (20.5)	3 (9.7)/6 (19.4)	0/2(25)	0.642
NYHA Class III/IV	14 (35.9)/14 (35.9)	9 (29)/11(41.3)	5 (62.5)/1(12.5)	0.169
Dyslipidemia	24 (61.5)	16 (51.6)	8 (100)	0.015
DM	13 (33.3)	11 (35.5)	2 (25)	0.694
CrCl, ml/minute	59.58 ± 28.38	74.78 ± 26.94	49.42 ± 26.02	0.022
COPD	3 (7.7)	1 (3.2)	2 (25)	0.101
Stroke	13 (33.3)	10 (32.3)	3 (37.5)	0.779
PVD	12 (30.8)	9 (29)	3 (37.5)	0.682
CAD	23 (59)	20 (64.5)	3 (37.5)	0.235
AF	9 (23.1)	6 (19.4)	3 (37.5)	0.355
Previousvalvular replacement surgery	1 (2.6)	1 (3.2)	0	0.607
Need for urgent aortic valvular intervention	1 (2.6)	0	1 (12.5)	0.205
Risk evaluation:				
EuroScore II	7.97 ± 8.53	6.74 ± 7.59	12.72 ± 10.73	0.077
STS Mortality	3.18 ± 2.81	2.97 ± 3.01	4.00 ± 1.81	0.364
STS Morbimortality	15.39 ± 7.94	13.96 ± 7.20	20.93 ± 8.72	0.025

*Data are presented as mean ± SD or n (%) as appropriate. NYHA, New York Heart Association heart failure classification; DM, diabetes mellitus; CrCl, creatinine clearance; COPD, chronic obstructive pulmonary disease; PVD, peripheral vascular disease; CAD, coronary artery disease; AF, atrial fibrillation (paroxysmal or permanent)*.

As shown in Table 2, evaluation of electrocardiographic parameters and echocardiographic characteristics before TAVR were analyzed. At baseline, there were no differences between the two groups based on mean aortic gradient, aortic valvular area, and valvular aortic area indexed to body surface area. Also, left ventricular ejection fraction (LVEF), left ventricular end-diastolic dimension (LVEDD), aortic root diameter (AO), left ventricular posterior wall thickness (PWT), and interventricular septum thickness (IVST) were comparable between the two groups. There was a larger amplitude of the T wave on lead I before TAVR in the patients with PPM implantation than in the patients without PPM implantation (with PPM 0.10 ± 0.17 mv vs. without PPM −0.07 ± 0.19 mv, *p* = 0.028). No other significant statistical differences were found between the two groups at baseline.

**Table 2:** Echocardiographic characteristics and electrocardiographic parameters.

	**Total** **(*n* = 39)**	**Without pacemaker (*n* = 31)**	**With pacemaker** **(*n* = 8)**	***p* value**
Mean aortic gradient (mmHg)	49.85 ± 21.54	52.90 ± 22.03	38.00 ± 15.37	0.081
Aortic valvular area (cm2)	0.67 ± 0.23	0.65 ± 0.24	0.73 ± 0.18	0.409
Valvular aortic area indexed to body surface area (cm2/m2)	0.38 ± 0.14	0.36 ± 0.14	0.42 ± 0.12	0.298
LVEF (%)	51.92 ± 13.05	52.16 ± 13.05	51.00 ± 13.87	0.826
LVEDD (mm)	52.15 ± 9.69	51.39 ± 9.46	55.13 ± 10.64	0.337
AO (mm)	20.10 ± 2.11	20.35 ± 2.14	19.13 ± 1.81	0.144
PWT (mm)	12.54 ± 2.21	12.52 ± 2.31	12.63 ± 1.92	0.903
IVST (mm)	13.44 ± 2.79	13.52 ± 3.04	13.13 ± 1.55	0.729
Heart Rate (bpm)	73.44 ± 15.72	72.65 ± 16.39	76.50 ± 13.27	0.543
Sinus	32 (82.1)	26 (83.9)	6 (75)	0.617
AF	4 (10.3)	2 (6.5)	2 (25)	0.180
Other atrial rhythm	2 (5.1)	2 (6.5)	0	0.461
Abnormal cardiac electric axis	22 (56.4)	17 (54.8)	5(62.5)	0.697
PR Interval (ms)	166.11 ± 47.28	162.21 ± 50.38	185.00 ± 21.64	0.289
1° AVB	5 (12.8)	4 (12.9)	1(12.5)	0.976
S1 (mv)	1.75 ± 0.83	1.80 ± 0.84	1.57 ± 0.78	0.477
S2 (mv)	2.47 ± 1.71	2.44 ± 1.87	2.56 ± 0.99	0.875
S3 (mv)	1.97 ± 1.27	1.98 ± 1.36	1.93 ± 0.92	0.930
R5 (mv)	2.49 ± 1.01	2.48 ± 1.00	2.54 ± 1.09	0.878
R6 (mv)	2.06 ± 0.86	2.05 ± 0.93	2.10 ± 0.59	0.882
I ST (mv)	−0.05 ± 0.07	−0.05 ± 0.08	−0.05 ± 0.04	0.915
aVL ST (mv)	−0.05 ± 0.07	−0.04 ± 0.08	−0.07 ± 0.05	0.326
V5 ST (mv)	−0.10 ± 0.12	−0.10 ± 0.14	−0.11 ± 0.07	0.847
V6 ST (mv)	−0.11 ± 0.12	−0.10 ± 0.13	−0.11 ± 0.06	0.865
T wave in lead I (mv)	−0.03 ± 0.20	−0.07 ± 0.19	0.10 ± 0.17	0.028
T wave in lead avL (mv)	−0.06 ± 0.17	−0.07 ± 0.16	−0.01 ± 0.23	0.322
T wave in lead V5 (mv)	−0.06 ± 0.45	−0.12 ± 0.46	0.17 ± 0.36	0.103
T wave in lead V6 (mv)	−0.04 ± 0.39	−0.05 ± 0.41	−0.002 ± 0.30	0.760
QRS Complex (ms)	104.13 ± 22.97	104.81 ± 24.50	101.50 ± 16.83	0.722
LBBB	3 (7.7)	3 (9.7)	0	0.360
RBBB	5 (12.8)	3 (9.7)	2 (25)	0.268
LAFB	2 (5.1)	1 (3.2)	1 (12.5)	0.372
QT interval (ms)	417.44 ± 56.34	418.58 ± 57.31	413.00 ± 55.91	0.807
cQT interval (ms)	456.21 ± 43.02	451.73 ± 44.97	473.50 ± 30.77	0.206

*Data are presented as mean ± SD or n (%) as appropriate. LVEF, left ventricular ejection fraction; LVEDD, left ventricular end-diastolic dimension; AO, aortic root diameter; PWT, left ventricular posterior wall thickness; IVST, interventricular septum thickness; AF, atrial fibrillation; 1° AVB, first degree atrioventricular block; S1, S wave magnitude in lead V1; S2, S wave magnitude in lead V2; S3, S wave magnitude in lead V3; R5, R wave magnitude in lead V5; R6, R wave magnitude in lead V6; I ST, ST-segment in lead I; aVL ST, ST-segment in lead aVL; V5 ST, ST-segment in lead V5; V6 ST, ST-segment in lead V6; LBBB, left bundle branch block; RBBB, right bundle branch block; LAFB, left anterior fascicular block; cQT interval, corrected QT interval*.

Table 3 shows the pattern of electrocardiographic changes in patients before and after TAVR. Our study did not identify any significant differences in the incidence of electrocardiographic conduction disturbances, such as new-onset atrial fibrillation (AF), new-onset 1st degree AV block, new-onset right bundle branch block (RBBB), or new-onset left anterior fascicular block (LAFB), with the exception of new-onset left bundle branch block (LBBB) (without PPM 22.6% vs. with PPM 75%, *p* < 0.001), as shown in Table 3.

**Table 3:** Procedural related electrocardiographic conduction disturbances.

	**Total (*n* = 39)**	**Without**	**With**	***p* value**
		**pacemaker**	**pacemaker**	
		**(*n* = 31)**	**(*n* = 8)**	
New-onset AF	7 (17.9)	5 (16.1)	2 (25)	0.617
New-onset 1° AVB	8 (20.5)	5 (16.1)	3 (37.5)	0.323
New-onset LBBB	13 (33.3)	7 (22.6)	6 (75)	<0.001
New-onset RBBB	4 (10.3)	4 (12.9)	0	0.284
New-onset LAFB	2 (5.1)	2 (6.5)	0	0.461

*Data are presented as mean ± SD or n (%) as appropriate. AF, atrial fibrillation; 1° AVB, first degree atrioventricular block; LBBB, left bundle branch block; RBBB, right bundle branch block; LAFB, left anterior fascicular block*.

**Figure 1:**
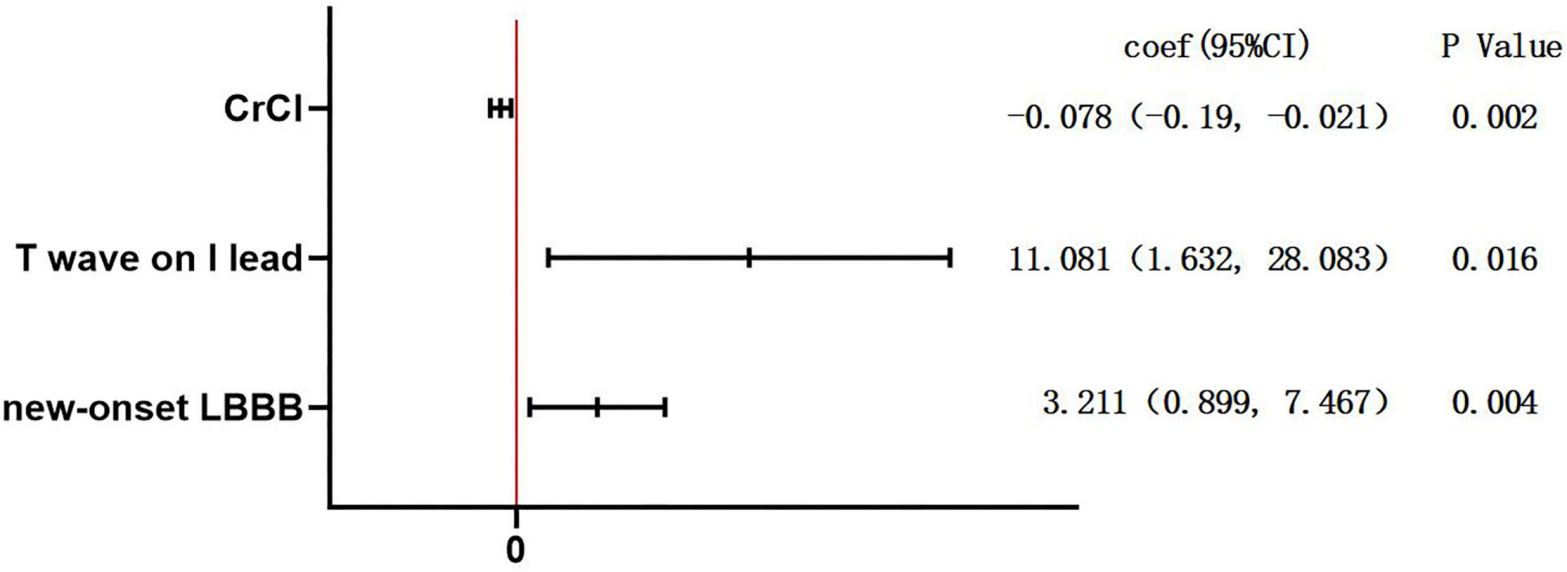
Multiple logistic regression analysis for the predictors of permanent pacemaker (PPM) implantation. To identify predictors of patients with PPM dependency after transcatheter aortic valve replacement (TAVR), logistic regression, with Firth's correction due to the small sample size, was performed. The variables to the right of the red line were predictors of PPM implantation. CrCl, creatinine clearance; LBBB, left bundle branch block.

Figure 1 summarizes the results involving predictors for patients having PPM implantation after TAVR. The main independent predictors of PPM implantation were new-onset LBBB (coef: 3.211, 95% CI: 0.899–7.467, *p* = 0.004) and T wave magnitude in lead I (coef: 11.081, 95% CI: 1.632–28.083, *p* = 0.016).

## Discussion

In the present study of 39 patients with self-expandable valves, we analyzed clinical data, echocardiographic characteristics, and electrocardiographic parameters. Four main findings of our study demonstrated that (1) the total rate of PPM implantation in our consecutive patients was 20.5%; (2) PPM implantation occurs with higher risk in patients with negative CrCl, dyslipidemia, high STS Morbimortality scores, and increased T wave magnitude in lead I; (3) TAVR induced several cardiac electrical changes, such as increased R wave and T wave changes in lead V5; and (4) new-onset LBBB and T wave magnitude in lead I were the main independent predictors of PPM implantation in patients undergoing TAVR.

### Indications for PPM Implantation

The most common indication for PPM implantation in those who underwent TAVR was complete AVB in our study, which had also been demonstrated previously (7, 8). Consistent with previous data, a sick sinus syndrome (37.5%), including relevant sinus and bradycardia, was a leading indication in patients who underwent PPM implantation (9).

The anatomic relationship between the aortic valve and the cardiac conduction system is the foundation of the conduction disturbances in patients undergoing TAVR. Anatomically, the proximity of this portion of the atrioventricular node (AVN), the bundle of His, and the membranous part of the interventricular septum makes it more susceptible to conduction disturbances. The left bundle branch is close to the bottom of the interleaflet triangle together with the location of the right coronary leaflets and non coronary aortic valve (10–12). The incidence of complete AVB after TAVR is due to a direct mechanical injury to the AVN and/or the left bundle branch that results in ischemia, edema, and hematoma according to necropsy studies (10). Due to the invention of a self-expandable valve possessing a subannular section, the release of the valve will produce radial force to the left ventricular outflow tract (LVOT). The implantation of a self-expandable valve may lead to conduction block (13). Therefore, it is crucial to improve the surgical manipulations among experienced surgeons in order to decrease the risk of complete AVB after TAVR.

### Abnormal Clinical Indications for PPM Implantation

Patients with a higher burden of PPM implantation following TAVR had a higher prevalence of abnormal clinical indications. Some abnormal clinical data before the procedure may be related to injury within the underlying conduction system during TAVR (11). Du et al. (12) demonstrated that patients with prior LBBB had a relatively higher risk of PPM implantation in the Chinese population. Gaede et al. (9) summarized that patients who already had aortic valve replacement prior to TAVR required a PPM implantation less frequently. Our study showed that severe AVS patients who required PPM following TAVR were likely to present with dyslipidemia and negative CrCl. STS Morbimortality also played an important role in evaluating patients with a high frequency of conduction disturbances after TAVR.

Previous studies have reported that renal dysfunction at baseline predicted a higher increase of myocardial injury after the procedure. Additionally, myocardial injury after TAVR could lead to conduction disturbances, which may contribute to the need for PPM implantation (14–16). Thus, lower average CrCl may lead to worse kidney function at baseline, which plays an important role in patients requiring PPM implantation after TAVR. Lindman et al. (17) showed that elevated blood lipid parameters were associated with higher risks of calcific AVS. On account of the thickening and remodeling of the aortic valve leaflets, the formation of calcific AVS will cause severe cardiac outflow tract stenosis. Prosthesis implantation may produce radial force to the cardiac outflow tract, which results in conduction block. It is therefore recommended that doctors focus more attention on reducing the risk factors before TAVR to decrease the need for subsequent PPM implantation.

### Predictors of PPM Implantation After TAVR

Few studies have evaluated the relationship between new-onset LBBB and the risk of PPM implantation after TAVR. In our cohort, a new-onset LBBB and T wave magnitude in lead I were the predictors for the patients requiring a PPM implantation after TAVR. However, prior RBBB at baseline was identified as an independent predictor in some previous studies (7, 11, 18, 19). In contrast to our study, smaller studies have shown that prior RBBB plays an important role in cardiac conduction system disorders after TAVR (8, 9, 20, 21).

The predictors of PPM implantation in patients receiving TAVR have been examined in seven studies (9, 12, 22–26). All studies were cohort studies. RBBB prior to TAVR was considered to be a predictor in most of the selected studies (9, 12, 22, 24, 26) in Table 4. However, the association of new-onset LBBB with PPM implantation after TAVR has not been fully characterized. A study by Chorianopoulos et al. (22), in which 46 patients with PPM implantation and 83 patients without PPM implantation were enrolled, showed that prior RBBB was the only predictor. A retrospective cohort study by Muillet et al. (23) showed that post-TAVR QRS duration and depth of implantation were the predictors of PPM implantation in patients receiving TAVR. In a cohort study by Luise Gaede et al. (9), in which 176 patients with PPM implantation and 849 patients without PPM implantation were enrolled, not only prior RBBB but also higher MPG and post-dilatation of the prosthesis were observed to be the predictors. A study focusing on 38 Chinese patients with PPM implantation and 218 Chinese patients without PPM implantation showed that prior RBBB, tricuspid aortic valve (TAV), and implantation depth at the non-coronary sinus side were the predictors, especially in Chinese population (12). The study of Kiani S, et al. (24) showed the history of syncope, prior RBBB, QRS duration ≥138 ms, and valve oversizing >15.6% were the predictors. Ferreira T et al. (25) demonstrated that increased H-V interval, which showed in post-TAVR electrophysiological study, was the best predictor of PPM implantation undergoing TAVR. A previous study by Johny Nicolas et al. (26) showed that RBBB before TAVR was the only predictor.

**Table 4:** Summary of studies regarding predictors of Permanent Pacemaker Implantation after TAVR.

**Authors**	**Year**	**Country**	**Study design**	**Groups**	**Numbers of subjects**	**Predictors**	**Ref**
Chorianopoulos et al. (22)	2012	Germany	Cohort	With PPM	46	prior RBBB	22
				Without PPM	83		
Mouillet et al. (23)	2013	France	Cohort	With PPM	21	Post-TAVR, QRS duration, Depth of implantation	23
				Without PPM	58		
Gaede et al. (9)	2017	Germany	Cohort	With PPM	176	prior RBBB, higher mean aortic gradient and post- dilatation of the prosthesis	9
				Without PPM	849		
Du et al. (12)	2019	China	Cohort	With PPM	38	prior RBBB, TAV, implantation depth at the noncoronary sinus side	12
				Without PPM	218		
Kiani et al. (24)	2019	America	Cohort	With PPM	57	history of syncope, prior RBBB, QRS duration ≥138 ms, valve oversizing >15.6%	24
				Without PPM	721		
Ferreira et al. (25)	2020	France	Cohort	With PPM	21	HV interval	25
				Without PPM	53		
Nicolas et al. (26)	2020	The Netherlands	Cohort	With PPM	132	prior RBBB	26
				Without PPM	790		

*PPM, permanent pacemaker; TAVR, transcatheter aortic valve replacement; TAV, tricuspid aortic valve; HV, His to ventricle*.

A positive correlation between PPM implantation after TAVR and new-onset LBBB was demonstrated in our study. However, no correlation was found between PPM implantation and prior RBBB or 1° AVB. We attribute the finding to mechanical injury that mainly affected the left bundle branch. Preexisting damage to the right bundle branch may lead to complete AVB when exposed to TAVR. The mechanism of the relationship between T wave magnitude in lead I and PPM implantation is unknown. Meanwhile, other predictors, including the type of the valve and the depth of prosthesis implantation, were not confirmed in our cohort (27–30). To date, no detailed guidelines for the management of new-onset LBBB following TAVR are available. Consequently, any recommendation about the management of new-onset LBBB after TAVR should be treated with caution and further investigation is warranted.

### Study Limitations

The limitations of our study are as follows. First, our study had a small sample size and a short follow-up time. The patients undergoing TAVR were also from a single center. The numbers of patients with PPM implantation and without PPM implantation were low, which limits the statistics of the study. However, the data were revised with Firth's correction due to the small sample size. All analyses were conducted using R (version 3.2.2; R Foundation for Statistical Computing, Vienna, Austria) or SAS Statistics (version 9.4; North Carolina, America). Thus, we strongly believe that our findings are reliable. Second, following discharge, we observed the patients only after a one-month follow-up. The interpretation may differ based on six-month or one-year follow-up periods. Finally, other variables, such as the position of the valve and valve type, may have generated a significant variation to the risk of conduction abnormalities after TAVR. Although our study cohort was small, only involved a single center, and was limited to a short follow-up time, we strongly believe that our findings are representative and may assist in providing valuable insight toward future identification of high-risk cases requiring PPM. Hence, our findings about PPM implantation after TAVR contributes to not only patients but also doctors for improving the postoperative quality of life of patients.

## Conclusions

The overall incidence of PPM implantation in our consecutive patients was 20.5%. PPM implantation occurs with higher risk in patients with negative CrCl, dyslipidemia, high STS Morbimortality score, and increased T wave magnitude in lead I. TAVR induced several cardiac electrical changes, such as increased R wave and T wave changes in lead V5. New-onset LBBB and T wave magnitude in lead I were the main independent predictors of PPM implantation in patients undergoing TAVR.

## Data Availability Statement

The raw data supporting the conclusions of this article will be made available by the authors, without undue reservation.

## Ethics Statement

The studies involving human participants were reviewed and approved by the Ethics Committee of Affiliated Zhongshan Hospital of Dalian University. The patients/participants provided their written informed consent to participate in this study.

## Author Contributions

JZ, CC, and ST: conceptualization and methodology. JZ and CC: investigation and writing—original draft. JZ: writing—review and English editing. ST, SZ, and JL: supervision. All authors contributed to the article and approved the submitted version.

## Conflict of Interest

The authors declare that the research was conducted in the absence of any commercial or financial relationships that could be construed as a potential conflict of interest.

## Publisher's Note

All claims expressed in this article are solely those of the authors and do not necessarily represent those of their affiliated organizations, or those of the publisher, the editors and the reviewers. Any product that may be evaluated in this article, or claim that may be made by its manufacturer, is not guaranteed or endorsed by the publisher.
