# Supplementary material for: Predictors of Permanent Pacemaker Implantation in Patients After Transcatheter Aortic Valve Replacement in a Chinese Population
Source: Front Cardiovasc Med. 2022 Jan 6;8:743257. doi: 10.3389/fcvm.2021.743257 (PMC8770941; doi:10.3389/fcvm.2021.743257)
Supplement: Supplementary file 1 [file Data_Sheet_1.PDF]

paitent	Male gend	Age, years	DM	Dyslipide	CrCl, ml/	PVD	AF	CAD
paitent1	Yes	60	No	Yes	126.36	No	No	Yes
paitent2	Yes	70	No	Yes	102.15	No	No	Yes
paitent3	Yes	65	No	No	85.21	Yes	No	Yes
paitent4	Yes	55	Yes	No	83.93	No	No	Yes
paitent5	No	83	No	No	45.07	No	Yes	No
paitent6	No	83	No	No	40.76	No	No	No
paitent7	No	79	Yes	No	60.93	No	No	Yes
paitent8	No	78	Yes	Yes	55.48	No	Yes	Yes
paitent9	Yes	75	Yes	Yes	65.83	No	No	No
paitent10	Yes	67	Yes	Yes	62.53	No	No	Yes
paitent11	Yes	82	Yes	No	65.21	No	No	Yes
paitent12	No	87	No	No	23.59	Yes	No	Yes
paitent13	Yes	85	No	Yes	84	No	No	Yes
paitent14	No	86	No	No	35.66	Yes	No	No
paitent15	Yes	68	No	Yes	88.98	Yes	No	Yes
paitent16	Yes	73	Yes	Yes	8.54	No	No	No
paitent17	No	62	No	No	102.15	No	No	No
paitent18	No	71	No	No	90.44	No	No	Yes
paitent19	Yes	77	Yes	Yes	72.29	No	Yes	No
paitent20	Yes	64	No	Yes	79.1	Yes	Yes	No
paitent21	Yes	82	No	No	68.28	No	Yes	Yes
paitent22	No	84	Yes	No	48.23	Yes	No	Yes
paitent23	Yes	66	No	Yes	118.21	Yes	No	No
paitent24	Yes	63	Yes	No	82.25	No	Yes	No
paitent25	Yes	84	Yes	Yes	46.1	No	No	Yes
paitent26	No	74	No	Yes	56.63	No	Yes	Yes
paitent27	Yes	73	No	Yes	36.07	No	No	No
paitent28	No	77	Yes	Yes	32.44	No	No	Yes
paitent29	Yes	82	No	Yes	51.56	Yes	Yes	Yes
paitent30	Yes	87	No	Yes	60.45	No	No	No
paitent31	Yes	69	No	Yes	141.62	Yes	No	Yes
paitent32	No	85	No	Yes	52.45	Yes	No	Yes
paitent33	Yes	71	No	Yes	98.85	No	No	Yes
paitent34	No	85	No	Yes	51.11	Yes	Yes	No
paitent35	No	72	No	Yes	98.63	Yes	No	No
paitent36	No	81	No	Yes	78.94	No	No	No
paitent37	No	78	No	No	89.87	No	No	No
paitent38	No	77	Yes	No	70.79	No	No	Yes
paitent39	No	65	No	Yes	52.84	No	No	Yes

Euroscore	STS morta	STS morbi	Baseline	Baseline	Baseline	Baseline	Baseline	Baseline
0.73	0.88	7.07	65	0.7	0.35	69	43	18
1.33	1.22	8.83	84	0.5	0.27	61	48	18
1.37	1.11	5.61	66	0.64	0.34	57	45	16
9.72	1.94	10.11	55	0.61	0.3	60	60	12
3.89	3.94	14.98	64	0.2	0.13	51	57	10
8.86	5.12	23.85	52	0.37	0.25	42	43	11
2.91	5.26	19.57	60	0.54	0.29	44	41	14
4.6	1.98	11.1	13	0.92	0.54	57	50	9
1.15	1.38	9.91	39	0.93	0.47	68	43	15
6.77	1.07	9.57	29	0.7	0.36	44	45	10
7.19	1.94	14.99	32	0.61	0.34	32	54	10
32.68	13.73	35.93	92	0.22	0.17	43	47	14
7.89	5.28	21.93	47	0.58	0.29	55	63	12
14.78	3.28	14.11	63	0.38	0.22	30	76	9
2.44	1	7.24	26	0.8	0.38	45	54	13
2.97	2.79	16	52	0.58	0.31	64	47	15
0.66	0.97	6.59	76	0.83	0.49	68	50	11
5.71	2.15	13.98	46	0.5	0.24	50	53	13
5.1	2.03	14.65	43	0.66	0.34	35	58	13
5.04	1.5	13.11	59	0.92	0.56	30	75	11
2.22	1.77	9.53	15	0.97	0.51	66	46	10
27.66	11.49	24.74	63	0.32	0.21	39	50	12
1.47	0.94	6.67	93	0.54	0.29	68	38	12
1.41	0.67	6.92	71	0.67	0.37	74	44	15
14.76	2.12	13.7	19	0.8	0.43	40	52	12
11.49	4.93	26.21	45	0.55	0.31	60	46	11
5.37	3.86	17.91	41	1.02	0.59	51	61	15
29.34	6.85	32.1	13	0.9	0.58	32	64	10
18.34	3.55	28.14	49	0.5	0.27	34	75	13
26.36	5.9	27.29	51	0.7	0.41	40	48	12
4.57	1.4	10.53	38	0.9	0.49	56	48	13
17.43	6.57	22.46	72	0.6	0.38	50	57	13
1.94	0.81	5.45	74	0.66	0.34	60	47	14
5.9	2.82	12.27	16	0.76	0.46	66	54	14
1.99	1.3	7.52	37	0.82	0.45	61	46	11
4.85	1.72	10.32	73	0.44	0.26	61	38	14
2.58	2.22	14.39	22	0.75	0.43	64	62	12
2.73	3.85	25.75	49	0.7	0.41	65	44	12
4.59	2.8	19.3	40	1.3	0.86	33	62	10

Baseline	Discharge	Discharge	Discharge	Discharge	Discharge	Discharge	Discharge	1M follow
18	7	1.8	0.9	68	42	18	18	12
21	35	1.12	0.6	61	50	15	16	27
20	18	1.43	0.76	65	46	15	16	30
12	19	1.83	0.89	59	59	12	12	12
11	10	1.88	1.24	59	57	10	11	8
12	6	1.12	0.75	56	43	11	12	5
14	39	1.03	0.56	55	47	11	15	29
10	5	1.4	0.83	60	50	9	10	4
15	8	1.69	0.85	62	45	12	13	11
10	13	1.53	0.79	50	52	10	12	9
11	5	1.2	0.66	38	54	10	11	7
15	24	0.99	0.78	50	45	14	15	18
12	9	2.07	1.05	51	58	12	12	8
9	14	2.2	1.28	33	66	10	10	12
15	10	2.57	1.24	46	54	13	15	10
15	10	1.4	0.75	56	45	15	17	15
12	39	1.01	0.6	69	47	11	11	30
14	9	2.25	1.1	55	50	13	14	7
13	27	1.39	0.72	40	57	13	13	23
10	28	1.62	0.99	37	73	11	10	27
11	7	2.24	1.16	55	44	10	11	6
14	10	0.94	0.63	41	45	12	14	9
15	23	1.14	0.61	70	38	12	15	25
15	16	2.13	1.18	55	45	12	12	18
12	19	1.55	0.83	50	60	11	11	14
11	16	1.31	0.74	54	48	14	12	17
15	26	1.61	0.93	50	59	15	14	14
11	7	1.5	0.97	40	64	10	10	8
13	9	1.2	0.65	40	74	10	15	12
13	14	1.34	0.78	48	47	12	13	11
15	10	1.38	0.75	52	49	12	16	9
16	14	1.04	0.67	58	54	13	16	13
18	19	1.91	0.99	62	47	14	18	15
14	9	1.27	0.77	66	54	12	14	12
13	13	1.27	0.7	63	46	11	13	12
16	12	1.43	0.84	63	39	14	14	12
12	17	1.49	0.85	58	54	12	12	11
12	22	1.09	0.64	65	42	12	12	17
9	17	1.23	0.81	36	57	10	9	15

1M follow	1M follow	1M follow	1M follow	1M follow	1M follow	Baseline	Baseline	Baseline
1.45	0.72	62	43	13	17	86	No	96
1.65	0.88	62	49	15	16	62	Yes	110
1.43	0.76	62	47	15	16	44	No	156
1.88	0.92	64	57	13	13	89	No	80
2.01	1.33	58	51	10	11	65	No	140
1.3	0.87	62	41	11	12	115	No	82
1.09	0.59	55	47	11	15	61	No	84
1.5	0.88	65	50	9	10	85	No	86
2.36	1.19	61	45	12	13	78	No	100
1.64	0.84	60	45	10	12	94	No	112
1.32	0.73	54	50	10	11	77	No	90
1	0.79	55	43	14	14	82	No	146
2.01	1.02	48	58	12	12	78	No	114
2.1	1.23	41	63	10	10	85	Yes	82
1.95	0.94	59	51	13	15	55	Yes	102
1.64	0.88	54	48	15	17	87	No	118
1.08	0.64	67	45	11	11	57	No	84
2.65	1.29	60	47	11	12	70	No	94
1.28	0.66	54	51	12	12	86	No	160
1.66	1.01	37	73	11	10	64	No	124
2.42	1.26	65	44	10	11	52	No	116
1.97	1.31	60	45	13	14	77	No	94
1.32	0.71	66	39	11	13	69	No	88
1.41	0.78	59	46	12	12	67	No	84
1.64	0.88	47	60	11	11	65	Yes	160
1.51	0.85	57	48	14	12	77	No	112
1.73	1	50	54	15	14	79	No	92
1.47	0.95	30	66	10	10	80	No	116
1.4	0.75	42	69	10	14	66	No	118
1.51	0.88	50	47	12	13	95	No	74
2	1.09	53	46	12	12	96	No	98
1.42	0.92	57	51	14	14	60	No	92
1.6	0.83	63	47	14	14	60	No	119
1.31	0.79	61	54	12	12	51	Yes	86
1.42	0.78	61	46	11	13	77	No	96
1.48	0.87	64	39	14	14	67	No	84
1.55	0.89	58	54	12	12	70	No	98
1.26	0.74	67	42	11	10	41	No	90
1.35	0.89	38	55	10	9	95	No	84

Baseline I ST	Baseline aVL S	Baseline V5 ST,	Baseline V6 S	Baseline	Baseline	Baseline
-0.2	-0.23	-0.15	0.1	No	No	No
0.1	-0.05	-0.2	-0.15	No	No	No
-0.7	-0.7	-1.5	-1.2	Yes	No	No
0.2	-0.2	0.2	0.15	No	No	No
0.1	0.1	0.48	0.4	No	Yes	No
-0.05	-0.05	0.1	0.05	No	No	No
-0.1	-0.1	-0.6	-0.4	No	No	No
-0.05	0	0.15	0.1	No	No	No
0.05	-0.1	-0.15	0.1	No	No	No
0.1	0.08	0.2	0.2	No	No	No
-0.15	-0.05	0.21	0.15	No	No	No
-0.2	-0.25	-0.2	0.2	Yes	No	No
-0.07	-0.04	-0.17	-0.23	No	No	No
0.05	0.03	0.18	0.15	No	No	No
0.02	-0.02	0.4	0.34	No	Yes	No
-0.18	-0.3	-0.2	-0.18	No	No	Yes
-0.04	-0.03	-0.19	-0.18	No	No	No
0.13	0.08	-0.12	0.09	No	No	No
-0.35	0.03	0.9	0.9	Yes	No	No
-0.1	-0.11	-0.8	-0.87	No	No	No
0.11	0.12	-0.1	0.22	No	No	No
-0.15	-0.17	-0.13	-0.24	No	No	No
-0.2	-0.14	-0.17	-0.09	No	No	No
-0.2	-0.15	-0.13	-0.15	No	No	No
0.03	0.1	-0.12	0.18	No	Yes	No
0.25	-0.3	0.7	0.35	No	No	No
0.02	-0.03	0.3	-0.2	No	No	No
0.22	0.37	0.22	0.04	No	No	No
0.2	0.18	-0.35	-0.4	No	No	No
0.15	0.05	0.25	0.18	No	No	No
0.18	0.08	0.35	0.36	No	No	No
-0.23	-0.17	-0.8	-0.7	No	No	No
-0.38	-0.24	-0.86	-0.74	No	No	Yes
-0.11	-0.09	-0.08	-0.23	No	Yes	No
0.25	0.08	0.5	0.42	No	Yes	No
-0.1	-0.05	-0.25	-0.17	No	No	No
0.2	0.06	0.25	0.21	No	No	No
-0.05	-0.01	-0.25	-0.15	No	No	No
-0.1	-0.12	-0.4	-0.2	No	No	No

Baseline	Baseline	Discharge	Discharge	Discharge	Discharge	Discharge	PPM
396	473	No	No	No	No	No	No
430	436	No	No	No	No	No	No
560	478	No	No	No	No	No	No
398	484	No	No	No	No	No	No
468	480	No	No	No	Yes	Yes	No
348	481	No	Yes	No	No	No	No
426	428	No	No	No	No	No	No
370	440	Yes	No	No	No	No	No
396	451	No	No	No	Yes	No	No
384	480	No	Yes	Yes	No	No	No
246	278	No	No	No	No	No	No
470	549	No	No	Yes	No	No	No
422	481	No	No	Yes	No	No	No
398	473	No	Yes	No	No	No	No
532	508	No	Yes	No	No	No	No
420	505	No	Yes	Yes	No	No	Yes
448	436	No	No	No	No	No	No
428	462	No	No	Yes	No	No	No
404	483	Yes	No	Yes	No	No	No
420	433	Yes	No	No	No	Yes	No
436	405	No	No	No	No	No	No
398	450	Yes	No	No	No	No	No
394	413	No	No	No	No	No	No
420	443	No	No	No	No	No	No
394	411	No	Yes	No	Yes	No	No
362	522	Yes	No	Yes	No	No	Yes
406	465	No	No	Yes	No	No	Yes
412	475	No	Yes	No	No	No	Yes
450	471	Yes	No	No	No	No	Yes
344	432	No	No	Yes	No	No	Yes
386	487	No	No	No	No	No	No
478	478	No	No	No	No	No	No
450	450	No	No	Yes	No	No	No
524	482	No	Yes	Yes	No	No	Yes
386	436	No	No	Yes	No	No	Yes
410	433	No	No	No	Yes	No	No
412	444	No	No	Yes	No	No	No
502	414	Yes	No	No	No	No	No
352	442	No	No	No	No	No	No
