# Supplementary material for: Predictors of Permanent Pacemaker Implantation in Patients After Transcatheter Aortic Valve Replacement in a Chinese Population
Source: Front Cardiovasc Med. 2022 Jan 6;8:743257. doi: 10.3389/fcvm.2021.743257 (PMC8770941; doi:10.3389/fcvm.2021.743257)
Supplement: Supplementary file 2 [file Data_Sheet_2.DOCX]

Electrocardiographic parameters in patients without PPM implantation after TAVR: before the procedure, at discharge and at one-month follow-up.

|  | | Time of Evaluation | | |  | p-value | |
| --- | --- | --- | --- | --- | --- | --- | --- |
|  |  | Baseline  (N=31) | Discharge  (N=31) | 1M follow-up  (N=31) |  | Baseline vs.  Discharge | Baseline vs. 1M  follow-up |
| Heart Rate (bpm) | | 72.65±16.39 | 81.65±15.97 | 74.61±13.58 |  | 0.018 | 0.556 |
| Sinus | | 26 (83.9) | 25 (80.6) | 25 (80.6) |  | 0.317 | 0.564 |
| AF | | 2 (6.5) | 5 (16.1) | 4 (12.9) |  | 0.083 | 0.157 |
| Other atrial rhythm | | 2 (6.5) | 1 (3.2) | 2 (6.5) |  | 0.317 | 1.000 |
| Abnormal cardiac electric axis | | 17 (54.8) | 20 (64.5) | 19 (61.3) |  | 0.366 | 0.593 |
| PR Interval (ms) | | 162.21±50.38 | 156.04±63.89 | 165.26±54.73 |  | 0.472 | 0.762 |
| 1° AVB | | 4 (12.9) | 5 (16.1) | 6 (19.4) |  | 0.564 | 0.317 |
| S1 (mv) | | 1.80±0.83 | 1.82±0.86 | 1.74±0.73 |  | 0.898 | 0.611 |
| S2 (mv) | | 2.45±1.87 | 2.40±1.17 | 2.12±1.16 |  | 0.859 | 0.283 |
| S3 (mv) | | 1.98±1.36 | 2.00±1.41 | 1.79±1.13 |  | 0.936 | 0.424 |
| R5 (mv) | | 2.48±1.00 | 2.20±1.06 | 2.03±1.09 |  | 0.128 | 0.024 |
| R6 (mv) | | 2.05±0.93 | 1.88±0.89 | 1.72±0.84 |  | 0.272 | 0.081 |
| I ST(mv) | | -0.05±0.08 | -0.09±0.16 | -0.04±0.07 |  | 0.262 | 0.755 |
| aVL ST (mv) | | -0.04±0.08 | -0.08±0.14 | -0.06±0.06 |  | 0.101 | 0.212 |
| V5 ST(mv) | | -0.10±0.14 | -0.10±0.13 | -0.06±0.13 |  | 0.878 | 0.100 |
| V6 ST(mv) | | -0.10±0.13 | -0.12±0.10 | -0.10±0.07 |  | 0.521 | 0.778 |
| T wave on I lead  (mv) | | -0.07±0.19 | -0.03±0.19 | -0.03±0.21 |  | 0.397  15 | 0.422 |
| T wave in aVL lead (mv) | | -0.07±0.16 | -0.08±0.20 | -0.09±0.16 |  | 0.783 | 0.568 |
| T wave in V5 lead (mv) | | -0.12±0.46 | -0.06±0.40 | 0.08±0.37 |  | 0.310 | 0.016 |
| T wave in V6 lead (mv) | | -0.05±0.41 | -0.15±0.37 | 0.01±0.38 |  | 0.230 | 0.437 |
| QRS Complex (ms) | | 104.81±24.50 | 110.90±26.07 | 110.61±30.46 |  | 0.164 | 0.187 |
| LBBB | | 3 (9.7) | 7 (22.6) | 6 (19.4) |  | 0.102 | 0.180 |
| RBBB | | 3 (9.7) | 4 (12.9) | 2 (6.5) |  | 0.564 | 0.317 |
| LAFB | | 1 (3.2) | 2 (6.5) | 2 (6.5) |  | 0.564 | 0.564 |
| QT | | 418.58±57.31 | 409.03±56.15 | 415.15±52.75 |  | 0.401 | 0.780 |
| QTc | | 451.74±44.97 | 469.68±49.44 | 458.61±35.64 |  | 0.075 | 0.394 |

Data are presented as mean ± SD or n (%) as appropriate. AF: atrial fibrillation; 1° AVB: first degree atrioventricular block; S1: S wave magnitude in V1 lead; S2: S wave magnitude in V2 lead; S3: S wave magnitude in V3 lead; R5: R wave magnitude in V5 lead; R6: R wave magnitude in V6 lead; I ST: ST-segment in I lead; aVL ST: ST-segment in aVL lead; V5 ST: ST-segment in V5 lead; V6 ST: ST-segment in V6 lead; LBBB: left bundle branch block; RBBB: right bundle branch block; LAFB: left anterior fascicular block; cQT interval: corrected QT interval.

Supplement 2 shows the electrocardiographic parameters of the patients in 31 patients without PPM before the procedure, at discharge and at one-month follow-up. At discharge, there was a significant increase in heart rate (baseline 72.65±16.39 bpm vs. discharge 81.65±15.97 bpm, p=0.018) which did not continue at one-month follow-up (74.61±13.58, p=0.556). Regarding the R wave and T wave changes in lead V5, there was a significant difference at one-month follow-up (R wave: baseline 2.48±1.00 mv vs. 1M follow-up 2.03±1.09 mv, p=0.024; T wave: baseline -0.12±0.46 mv vs. 1M follow-up 0.08±0.37 mv, p=0.016 ), contrary to the difference at discharge(-0.12±0.46, p=0.310).

16
